# Application of ultrasound measurement of optic nerve sheath diameter to guide hyperosmolar therapy in children with intracranial hypertension

**DOI:** 10.3389/fped.2025.1632992

**Published:** 2025-09-25

**Authors:** Yanping Hua, Jiawei Wang, Yong Li, Yuhao Shen, Leihua Jiang, Jiaowei Wu

**Affiliations:** Pediatric Intensive Care Unit (PICU), Affiliated Children’s Hospital of Jiangnan University (Wuxi Children’s Hospital), Wuxi, Jiangsu, China

**Keywords:** optic nerve sheath diameter, increased ICP, osmotherapy, pediatric, pediatric intensive care unit

## Abstract

**Purpose:**

Assessing the Clinical Value of Optic Nerve Sheath Diameter (ONSD) Ultrasonography in Pediatric Severe Intracranial Hypertension Monitoring and Treatment Efficacy Evaluation.

**Methods:**

This study included 86 critically ill children with intracranial hypertension, and used bedside ultrasound to dynamically monitor the diameter of the optic ONSD to evaluate the status of intracranial hypertension. The experimental group (*n* = 33) underwent three daily ONSD ultrasound monitoring throughout the treatment process, with a baseline value of >5.2 mm set as the intervention threshold based on the guidelines of the American Society for Neurocritical Care; The control group (*n* = 53) was monitored using traditional clinical signs. The efficacy evaluation was conducted using the National Institute of Health Stroke Scale (NIHSS), and core indicators such as the duration of neurological function recovery, Intensive Care Unit (ICU) hospitalization period, and incidence of complications were comprehensively compared between the two groups of children.

**Result:**

ONSD measurements, hyperosmolar agent [mannitol, hypertonic saline(HTS)] were adjusted accordingly. The treatment group demonstrated significantly shorter duration of hyperosmolar agents compared to controls. Although the ultrasound-guided group showed reduced hospitalization duration relative to the control group, this difference did not reach statistical significance. Neurological outcomes evaluated by discharge Glasgow Coma Scale (GCS) scores revealed clinically meaningful differences: the treatment group exhibited higher proportion of fully conscious patients and lower incidence of consciousness with both parameters showing statistical significance.

**Conclusion:**

The implementation of optimized neurological intensive care protocols incorporating multimodal monitoring demonstrates significant prognostic benefits. ONSD measurement offers clinical advantages as a rapid, non-invasive modality for detecting intracranial pressure fluctuations, establishing its utility in therapeutic monitoring for pediatric patients with severe intracranial hypertension.

## 1 Introduction

Acute neurological emergencies in pediatric populations predominantly stem from multifactorial etiologies including traumatic brain injury, hypoxic-ischemic insults, and CNS infections (1). These pathologies induce cytotoxic edema and vasogenic edema through mechanisms involving blood-brain barrier disruption and cellular energy failure, culminating in intracranial hypertension with secondary complications including cerebral herniation (2). Ultimately, this pathophysiological cascade may progress to life-threatening conditions or irreversible neurological deficits in untreated cases.

A multinational study analyzing data from over 165,000 pediatric traumatic brain injury (TBI) cases across five continents reveals substantial geographical variation in reported incidence rates, ranging from 47–280 cases per 100,000 children globally (3). These epidemiological findings confirm traumatic brain injury as a significant pediatric public health concern. The immaturity of the developing immune system and blood-brain barrier in pediatric populations confers heightened vulnerability to intracranial infections, particularly in cases progressing to necrotizing encephalopathy—a condition associated with mortality rates of 30%–70% and complete neurological recovery observed in only 0%–5.7% of cases. Survivors frequently experience permanent neurological sequelae (4).

Pathophysiologically, both cytotoxic mechanisms and vascular-mediated cerebral edema contribute to elevated intracranial pressure (ICP), which subsequently compromises cerebral perfusion through reduced cerebral blood flow and diminished perfusion pressure (5). Current clinical management prioritizes timely ICP control as a critical determinant of therapeutic outcomes. While invasive ICP monitoring via implanted probes remains the diagnostic gold standard, its application in pediatric practice is limited by procedural invasiveness and substantial costs. Alternative methods such as lumbar puncture for cerebrospinal fluid pressure assessment prove unreliable in children due to measurement variability induced by agitation and the inability to perform repeated dynamic measurements (6). Notably, there exists limited evidence regarding optimal protocols for precise osmotherapy guided by dynamic ICP monitoring in pediatric intracranial hypertension management.

In recent years, non-invasive ICP monitoring methods have been actively explored in clinical practice. As the cerebrospinal fluid in the subarachnoid space of the optic nerve is connected to the intracranial subarachnoid space, the ONSD can indirectly reflect the level of ICP (7, 8). This article will use bedside ultrasound to measure the diameter of the optic nerve sheath, evaluate intracranial pressure by observing its dynamic changes, guide hyperosmolar therapy, and analyze ofclinical efficacy and application value.

## 2 Subjects and methods

### 2.1 Research object and grouping

#### 2.1.1 Study population

Pediatric patients presenting with intracranial hypertension were prospectively enrolled from the Pediatric Intensive Care Unit of Jiangnan University Affiliated Wuxi Children's Hospital between January 2023 and December 2024. Inclusion criteria comprised: (1) traumatic brain injury (TBI); (2) non-traumatic intracranial hemorrhage; (3) confirmed intracranial infection; (4) completion of cranial CT within 6 h post-admission; (5) minimum hospitalization duration of 5 days. Exclusion criteria included: (1) ocular trauma or optic nerve injuries; (2) pre-existing ophthalmic pathologies (e.g., orbital masses, optic neuritis); (3) intracranial space-occupying lesions; (4) treatment discontinuation or self-discharge.

#### 2.1.2 Cohort characteristics

The final cohort comprised 86 patients (male: *n* = 48; female: *n* = 38) aged 0–15 years (mean age 5.18 ± 3.34 years). Glasgow Coma Scale (GCS) scores at admission were distributed as follows: severe impairment (GCS 3–8, *n* = 50), moderate impairment (GCS 9–11, *n* = 20), mild impairment (GCS 12–14, *n* = 6), and normal consciousness (GCS 15, *n* = 10). Etiological classification included traumatic/non-traumatic hemorrhage (*n* = 44) and infectious causes (*n* = 42).

#### 2.1.3 Ethical considerations & study design

This investigation received approval from the Institutional Review Board (IRB-2023-018), with written informed consent obtained from all legal guardians. Based on institutional protocol evolution, the control group (*n* = 53) consisted of patients admitted prior to July 2023 who did not undergo optic nerve sheath diameter (ONSD) monitoring, while the measurement group (*n* = 33) included patients receiving standardized ONSD assessments from January 2024 onward. Demographic and baseline clinical comparisons between groups are detailed in Table 1.

**Table 1:** Case data from the ONSD group and control group.

Part	ONSD	Control	*χ*2/t	*P*
	(*n* = 33)	(*n* = 53)		
Male	18 (37.50)	30 (62.50)	0.035	0.852
Female	15 (39.47)	23 (60.53)		
Ages (X ± s)	5.06 ± 2.97	5.26 ± 3.57	0.243	0.292
Infection intracranial (%)	13 (39.4)	29 (54.7)	1.911	0.189
Intracranial hemorrhage (%)	20 (60.6)	24 (45.3)		
GCS	8.03 ± 3.35	8.15 ± 3.58	0.423	0.517
PCIS^*^	89.33 ± 9.9	85.91 ± 9.8	0.100	0.752

*PCIS: is a physiological scoring system developed in 1995 to assess the severity of critical illness in children (Chinese Pediatric Association formulate). It evaluates 10 parameters: Vital signs: Heart rate, blood pressure, respiratory rate, PaO₂ Internal environment: pH, Na^+^, K^+^, creatinine/urea nitrogen; Organ function: Hemoglobin, gastrointestinal status. Each parameter is scored 0–2 (total range: 0–20, converted to a 100-point scale). Severity categories: Non-critical: >80; Critical: 71–80; Extremely critical: ≤70.

### 2.2 Ultrasonic measurement of optic nerve sheath diameter

#### 2.2.1 ONSD measurement protocol

All measurements were performed within 6 h post-admission using a Mindray M9 portable ultrasound system. Subjects were positioned with:

1. 20–30° head elevation (contraindicated in cervical spine injuries)
2. Neutral head alignment
3. Protective sterile adhesive film over closed eyelids (preventing gel contact)

**Figure 1:**
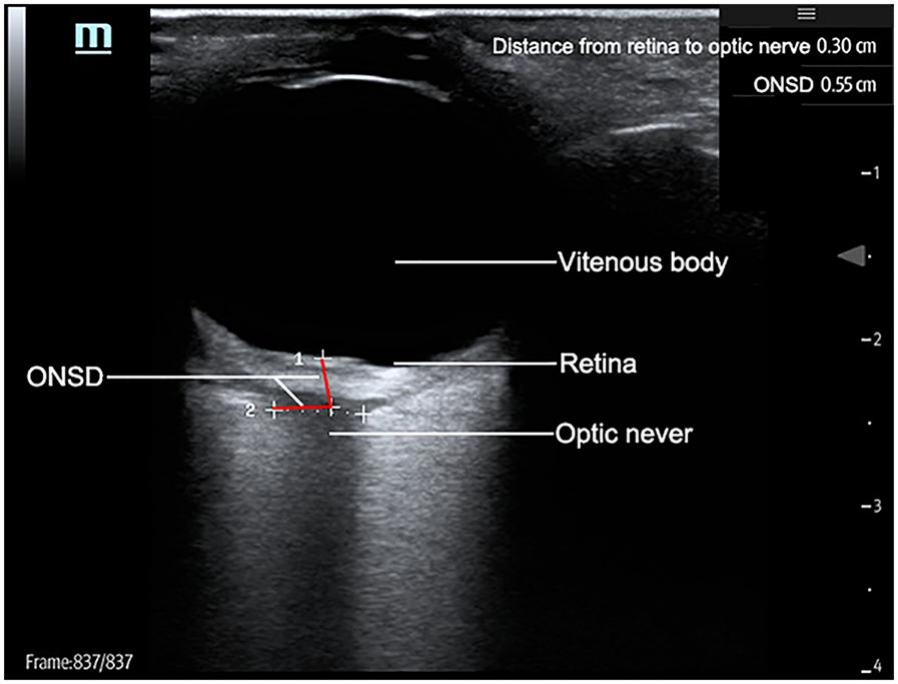
Diagram of optic nerve sheath in eyeball B-ultrasound.

A 7.5 MHz linear transducer was applied without compressive force on the eyelid. Optic nerve sheath diameter (ONSD) was measured in both axial and sagittal planes at 3 mm posterior to the globe. Each plane underwent duplicate measurements per eye, with the mean value recorded. Figure 1 illustrates the standardized measurement protocol.

### 2.3 Intracranial hypertension management

Both cohorts received tiered osmotherapy per expert consensus guidelines:

Acute phase (≤24 h post-TBI/hemorrhage): 3% hypertonic saline (5 ml/kg bolus).
Cerebral herniation: 20% mannitol (0.5–1 g/kg).
Infectious etiology: Mannitol priming followed by glycerol fructose (0.5 ml/kg q6–8 h).

Continuous physiological monitoring guided subsequent interventions including:

1. Maintenance of serum sodium 145–155 mmol/L
2. Cerebral perfusion pressure >40 mmHg
3. Euvolaemia (CVP 5–8 cmH2O)

### 2.4 Pediatric critical illness score(PCIS)

PCIS is a 1995-developed physiological scoring system for assessing pediatric critical illness severity. It evaluates 10 parameters across three domains: (1) Vital signs: HR, BP, RR, PaO₂; (2) Internal environment: pH, Na^+^, K^+^, creatinine/BUN.

Organ function: Hb, GI status; (3) Scored 0–2 per parameter (total 0–20, converted to 0–100 scale). Severity classification:

Non-critical: >80
Critical: 71–80
Extremely critical: ≤70.

### 2.5 Diagnosis of intracranial infection

#### 2.5.1 Clinical presentation

Typical symptoms include fever (>38 °C) with chills, severe headache and projectile vomiting, positive meningeal signs (nuchal rigidity), and altered consciousness (lethargy, coma) or psychiatric symptoms. Neurological deficits may manifest as focal neurological deficits (hemiparesis, aphasia), seizure episodes, or papilledema.

#### 2.5.2 Laboratory findings

CSF analysis (gold standard) shows elevated pressure (>200 mmH₂O), turbid appearance with WBC >100 × 10⁶/L (polymorphonuclear >70%), decreased glucose (<2.6 mmol/L), and elevated protein. Positive pathogen identification confirms diagnosis. Blood tests may reveal leukocytosis (>10 × 10⁹/L), neutrophilia (>80%), and elevated CRP.

#### 2.5.3 Imaging studies

CT/MRI features include meningeal enhancement (contrast-enhanced), cerebral edema or focal lesions, and abscess formation (ring enhancement). EEG may show abnormal waveforms (spikes, sharp waves).

#### 2.5.4 Diagnostic grading

Clinical diagnosis requires meeting clinical criteria plus imaging and CSF abnormalities (items 1–4).

Microbiological confirmation requires meeting clinical diagnosis plus positive culture (items 1–5).

### 2.6 Statistical analysis

Data analysis was conducted using SPSS 29.0 software. The measurement data is expressed as Mean ± SD. For those that follow a normal distribution, *t*-test is used for inter group comparison, and non parametric rank sum test is given for those that do not follow a normal distribution; Perform Fisher's test on qualitative data using examples (percentages). Perform correlation analysis using Spersman. The difference is statistically significant with *P* < 0.05.

## 3 Results

### 3.1 Comparison of general information between two groups of children

Among the 86 pediatric patients, they were divided into a treatment group and a control group based on whether ultrasound was used to evaluate ONSD. Both groups had neurological symptoms; The age group used was 5.06 ± 2.97, with 18 males and 15 females. The control group had an average age of 5.26 ± 3.57, with 30 males and 23 females; The classification of the two disease groups is similar, with intracranial infections accounting for 39.4% and intracranial hemorrhagic diseases (including traumatic and non traumatic) accounting for 60.6% in the use group, and the former accounting for 29% and the latter accounting for 24% in the control group; The severity of the two groups of diseases was evaluated by GCS score, pediatric critical illness score. The GCS score of the treatment group was 8.03 ± 3.35, the pediatric critical illness score was 89.33 ± 9.90, and the IL-6 value was 195.25 ± 150.55. The GCS score of the control group was 8.15 ± 3.58, the pediatric critical illness score was 85.91 ± 9.87. There was no statistically significant difference in the above general data comparison (*P* > 0.05). See Table 1.

### 3.2 Comparison of treatment outcomes

#### 3.2.1 Treatment outcomes analysis

Comparative analysis revealed significant differences in therapeutic parameters between the ONSD-guided intervention group (*n* = 33) and conventional treatment controls (*n* = 53). Key findings included.

##### 3.2.2.1 Osmotherapy duration

ONSD group: 4.55 ± 1.66 days (mean ± SD).

Control group: 7.38 ± 3.81 days.

(*p* < 0.01, Cohen's d = 1.12 indicating large effect size).

##### 3.2.2.2 Hospitalization length

ONSD group demonstrated reduced hospitalization (9.39 ± 3.49 days vs. 11.79 ± 5.05 days), though not statistically significant (*p* = 0.056).

##### 3.2.2.3 Neurological outcomes

Consciousness recovery at discharge showed clinically meaningful improvements:

Full Recovery (GCS ≥ 15): 75.8% (25/33) in ONSD group vs. 50.9% (27/53) in controls (*χ*² = 5.416, *p* = 0.02).
Persistent Impairment (GCS < 15): 15.2% (5/33) vs. 39.6% (21/53) (*χ*² = 4.12, *p* = 0.042).
Mortality: Comparable rates at 9.0% (3/33) vs. 9.5% (5/53) (*p* = 0.958).

#### 3.2.2 Clinical implications

The ONSD-guided protocol reduced osmotherapy exposure by 38.3% while achieving superior neurological recovery, suggesting improved cerebral perfusion pressure management without compromising safety (Table 2).

**Table 2:** Results of treatment in the ONSD group and control group.

Part	ONSD	Control	χ^2^/t	*p*
Treatment days	4.55 ± 1.66	7.38 ± 3.81	4.027	0.001
Hospitalization days	9.39 ± 3.49	11.79 ± 5.05	2.389	0.056
Mortality^a^	3 (9.0)	5 (9.5)	0.003	0.958
*N*	25 (75.8%)	27 (50.9%)	5.416	0.020

^a^ Mortality: We found that the deaths in the study were not significantly related to changes in intracranial pressure based on relevant case information.

### 3.3 Effects of dehydration treatment on ONSD

**Figure 2:**
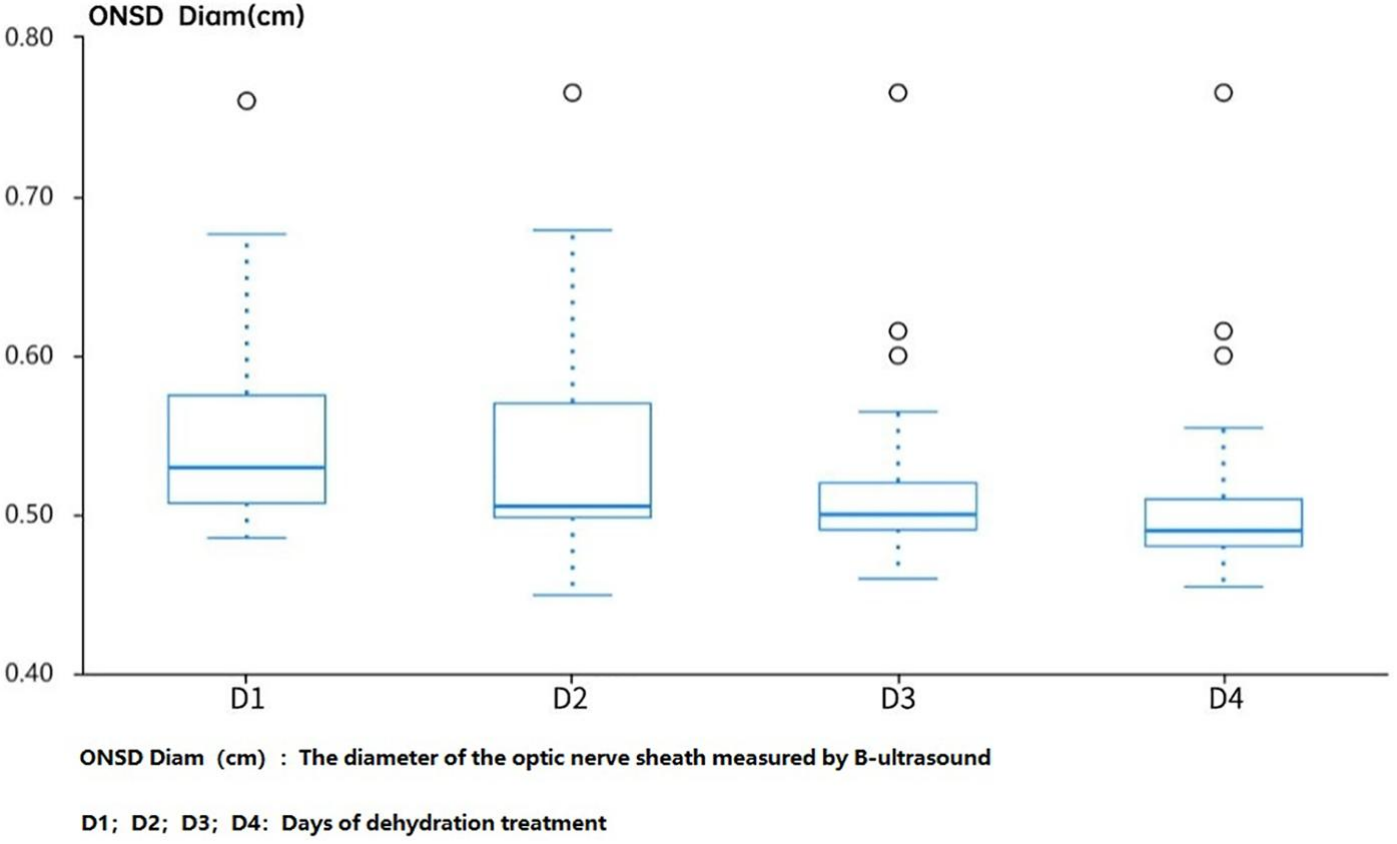
Dynamic changes of ONSD before and after dehydration treatment in the use group.

Observing the changes in ONSD before and after dehydration treatment in the ultrasound group on the first day of hospitalization, as well as 1 h, 2 h, and 4 h after medication, it was found that there was a significant change in ONSD after the use of dehydrating agents on the first day of hospitalization. However, on the fourth day, the diameter of ONSD decreased compared to admission, and the changes in ONSD decreased after application, suggesting that continuing to use dehydration treatment may not benefit much (Figure 2).

## 4 Discussion

ICP leading to a decrease in cerebral perfusion pressure is an important cause of secondary brain injury. Whether it is cytotoxic brain edema caused by infection or vascular brain edema caused by trauma or bleeding, ICP can increase to varying degrees. Increased ICP can cause cerebral herniation and consciousness disorders in children (9, 10). Hyperosmolar therapy is required during treatment to reduce intracranial pressure and ensure cerebral perfusion. Therefore, dynamic monitoring of intracranial pressure has become an important basis for clinical management (11). Under high intracranial pressure, pressure is transmitted through the subarachnoid space to the optic nerve sheath for dilation, which may be similar to the pathophysiological mechanism of optic nerve papilledema after high intracranial pressure (12). In recent years, ultrasound measurement of optic nerve sheath diameter has been increasingly used in the field of adult neurological critical illness for indirect intracranial pressure assessment Most studies have shown that the critical value for diagnosing increased ICP is 5.0–5.9 mm (12, 13). There is currently no clear standard for children, and more clinical data is needed to determine whether ONSD is related to factors such as age and development. In a prospective cohort study of pediatric neurosurgical patients, Kerscher et al. (2022) demonstrated significant age-dependent correlations between optic nerve sheath diameter (ONSD) and intracranial pressure (ICP). Their findings revealed: Overall correlation: Strong association (r = 0.78, *p* < 0.001) between ONSD and ICP across all ages. This discrepancy was attributed to the compensatory role of patent fontanelles in attenuating ICP elevations in infants, as open cranial sutures allow for cranial vault expansion during intracranial hypertension episodes (14). At the same time, it was pointed out that the optimal cut-off values for ONSD values in ICP > 15 mmHg and > 20 mmHg are 5.28 and 5.57 mm, respectively. Steinborn et al. measured the diameter of the optic nerve sheath in children and adolescents without neurological or ophthalmic diseases using magnetic resonance imaging and ultrasound, and found that the measured values were all higher than 5 mm, indicating that there is a certain degree of difference between children and adults with ONSD. The average age of the enrolled children in this study was 5 years old, and children under 1 year old were not included to avoid the impact of fontanelle on ONSD (15).

At present, there is sufficient evidence to show the correlation between ultrasound measurement of ONSD and ICP in traumatic brain injury, and it has been included in clinical guidelines for TBI management in some European and American countries (16, 17). Madhura B et al. conducted a systematic review of the relevant literature and pointed out that ONSD has a good correlation with changes in ICP in intracranial infection, stroke, cerebrovascular accidents, and other diseases (18). There are also studies that have guided the prediction of ICP in intracranial infection patients at admission and around 2 weeks of follow-up (19).

Although there is currently no unified standard for children's ONSD, an increase in ICP is positively correlated with ONSD, and the diameter of the optic nerve sheath also changes with the improvement of ICP (20). Meanwhile, Giulia Abbinante et al. also confirmed this in a recent literature review (21). Therefore, the dynamic changes of ICP can be obtained by monitoring ONSD. There was no significant statistical difference in the general admission situation, including GCS score and critical illness score, between the two groups of children in this study (see Table 1), while the dehydration treatment days in the group assessed by ultrasound were significantly shorter than those in the control group. The treatment outcomes of the two groups showed no statistically significant difference in mortality rate, but the proportion of awake children in the treatment group was higher than that in the control group, indicating that monitoring the optic nerve sheath before and after treatment in children with intracranial hypertension to understand changes in intracranial pressure and adjusting dehydration treatment plans in a timely manner can more effectively improve intracranial hypertension and thus improve prognosis.

A study conducted by Anggia F et al. on the use of bedside ultrasound to assess osmotic therapy in children with intracranial infections showed that there were significant changes in obstructive sleep apnea after using osmotic therapy (22). Bedside ultrasound can timely evaluate intracranial pressure, providing assistance in improving prognosis. As this study lacked dedicated efficacy analysis, we consulted the 2024 systematic review by Martínez Palacios et al. on ONS-based ICP determination, which referenced studies with sample sizes of 50–100 participants (8). To enhance methodological rigor, future research should incorporate targeted efficacy analyses. Should additional experimental data become available, such analyses will be prioritized.

This study has several methodological limitations that warrant discussion. First, the assessment protocol could be enhanced by incorporating additional critical evaluation parameters. Second, while ONSD measurements offer a non-invasive alternative for intracranial pressure estimation, the inherent operator-dependent variability of this technique was not rigorously assessed. Furthermore, the correlation analyses could benefit from more granular stratification, and a direct comparison with lumbar puncture pressure measurements—considered the clinical gold standard—was notably absent from the study design. Selecting research subjects clustered around July 2023 enables a clear observation of pre- and post-intervention changes following policy/technical implementations. However, this approach entails three potential biases: (1) interference from institutional variables, (2) case composition bias, and (3) confounding factors. This study has technical limitations, including the potential for measurement errors due to performing ultrasound examinations with closed eyes (23, 24). Additionally, incorporating standardized A-scan examinations would enhance the scientific rigor of this research (25).

Therefore, reducing dehydration treatment days alone may not necessarily shorten the entire course of treatment.

## 5 Conclusion

Optimizing monitoring oriented neurological intensive care management is beneficial for achieving better clinical outcomes, especially with the advantage of rapid, non-invasive, and effective reflection of intracranial pressure changes in bedside ultrasound monitoring of ONSD. It is expected to become an important indicator for early evaluation of clinical prognosis in patients with severe traumatic brain injury. Due to the small sample size and short follow-up time of this study, as well as subjective bias in the use of ultrasound to measure ONSD, further research is needed to verify the relevant conclusions.

## Data Availability

The original contributions presented in the study are included in the article/Supplementary Material, further inquiries can be directed to the corresponding author.
